# Formation and Detection of High-Pressure Oxygen in Closed Pores of La_0.6_Sr_0.4_CoO_3−δ_ Solid Oxide Electrolysis Anodes

**DOI:** 10.1021/acsaem.2c00888

**Published:** 2022-06-23

**Authors:** Martin Krammer, Alexander Schmid, Matthäus Siebenhofer, Andreas Ewald Bumberger, Christopher Herzig, Andreas Limbeck, Markus Kubicek, Juergen Fleig

**Affiliations:** † TU Wien, Institute of Chemical Technologies and Analytics, Getreidemarkt 9/164-EC, 1060 Vienna, Austria; ‡ Centre for Electrochemical Surface Technology GmbH, Viktor-Kaplan-Straßze 2, 2700 Wiener Neustadt, Austria

**Keywords:** solid oxide electrolysis cell (SOEC), oxygen electrode, chemical capacitance, LSC, anodic polarization, thin film

## Abstract

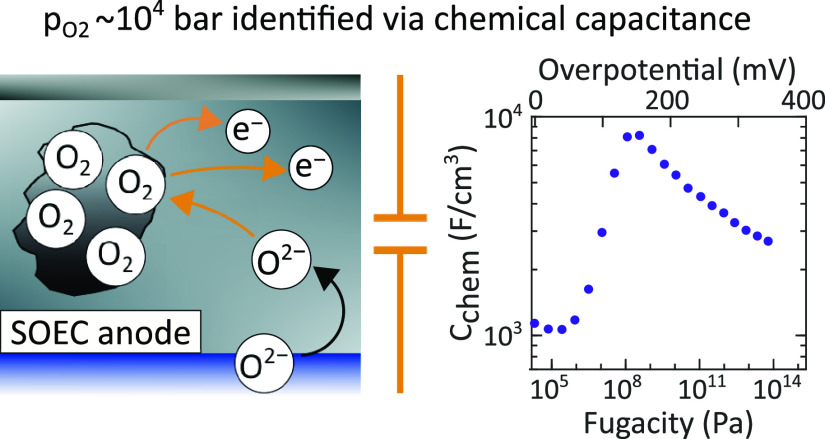

The chemical capacitance of La_0.6_Sr_0.4_CoO_3−δ_ (LSC) thin film microelectrodes with different microstructures was investigated upon varying anodic DC voltages. Dense and porous electrodes (open porosity) were prepared by using different parameters during pulsed laser deposition (PLD). Furthermore, electrodes with closed porosity were fabricated by depositing a dense capping layer on a porous film. Electrochemical impedance spectroscopy (EIS) was performed in synthetic air at 460 and 608 °C with anodic DC voltages up to 440 mV. Chemical capacitance values of the electrodes were derived from the obtained spectra. While the chemical capacitance of dense and porous electrodes decreased as expected with increasing anodic overpotential, electrodes with closed pores exhibited very unusual peaks with extremely high values of >8000 F/cm^3^ at overpotentials of >100 mV. We demonstrate that this huge capacitance increase agrees very well with calculated chemical capacitances deduced from a real gas equation. Hence, we conclude that the formation of highly pressurized oxygen (up to gas pressures of ∼10^4^ bar) in closed pores is responsible for this strong capacitive effect at anodic overpotentials. Such measurements can thus detect and quantify the buildup of high internal gas pressures in closed pores at the anode side of solid oxide electrolysis cells.

## 1 Introduction

The energy sector has to face drastic changes if the ever-growing global demand for energy shall be met with zero- or low-emission technologies. This change will certainly include a high share of renewable electricity production from wind and solar power. However, these technologies suffer from problems in matching supply and demand as their energy production fluctuates on a daily, weekly, and seasonal basis, which is why they are also referred to as variable or intermittent renewable energy sources.^1−5^ Therefore, sector coupling as well as energy-storage systems may play an important role in the future energy supply, and hydrogen technologies could become a key solution in both of these areas.^1−3,5−7^ Especially, solid oxide electrolysis cells (SOECs) may have a significant impact for both storage and sector coupling because of their potential for highly efficient production of hydrogen.^8−11^ Combined with solid oxide fuel cells (SOFCs), high round trip system efficiencies up to about 44% can be achieved (assuming 10% energy loss by compressing hydrogen for storage purposes^12^).^9,13^ Moreover, SOECs can be used for low-emission production of syngas or CH_4_ via carbon capture from fossil-fuel-fired plants and coelectrolysis of water steam and carbon dioxide.^14,15^

Nevertheless, SOECs need to overcome obstacles concerning performance degradation and stability of stack and cell components.^16−23^ Although the materials used in SOECs are similar to those in SOFCs, degradation phenomena can be quite different because of inverse operating conditions. Particularly, microstructural and morphological changes at the oxygen electrode (anode) side occurring under electrolysis operation (i.e., anodic polarization) are a common problem, leading to severe performance deterioration. Postoperation analyses revealed pore and crack formation in the electrolyte material^23,24^ or at the anode/electrolyte interface^22,25,26^ as well as delamination of the anode from the electrolyte or the barrier layer.^19,24,25,27−31^ These degradation phenomena were reported for cells using yttria-stabilized zirconia (YSZ) and La_1–*x*_Sr_*x*_MnO_3−δ_ (LSM) or La_1–*x*_Sr_*x*_Co_*y*_Fe_1–*y*_O_3−δ_ (LSCF), which are the most common SOEC materials for the electrolyte and the oxygen electrode, respectively. Several reports suggested that the buildup of high internal gas pressures in closed pores of the electrolyte, the anode, or at the anode/electrolyte interface causes mechanical stress and is thus responsible for the above-described degradation phenomena.^19,25,28,31−33^ So far, the values of these gas pressures have been estimated on the basis of the corresponding overpotential,^23,31−34^ however considering their potentially detrimental effects, a method which directly quantifies these pressures would be highly desirable.

Here, we propose a novel approach to determine these internal gas pressures which includes the analysis of the oxygen electrode’s chemical capacitance. The chemical capacitance of an oxide *C*_chem_ is typically used to obtain information about the defect chemistry^35−37^ and is then defined as follows^38,39^
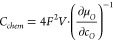
1where *F* denotes the Faraday constant and *V* the electrode’s (bulk) volume. Thus, the chemical capacitance scales with the volume and is determined by the derivative of the oxygen chemical potential μ_O_ with respect to the concentration of oxygen *c*_O_. This approach can be extended to the gas phase, present in a given volume *V* via
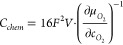
2with the chemical potential and concentration of O_2_ being  (= 2 μ_O_) and  (= *c*_O_/2), respectively. Since the derivative depends on the oxygen partial pressure, the chemical capacitance may be used for the determination of internal gas pressures.

This is demonstrated for La_0.6_Sr_0.4_CoO_3−δ_ (LSC) thin film electrodes with closed pores operated upon anodic polarization (corresponding to the SOEC mode). Closed porosity was introduced by depositing a dense capping layer on top of a porous thin film using pulsed laser deposition. Very unusual peaks of the chemical capacitance resulted from impedance measurements, with values exceeding 8000 F/cm^3^ at overpotentials higher than 100 mV. It turned out that explaining these capacitive peaks is nontrivial since it requires to include a real gas equation, yielding extremely high gas pressure and fugacity values. However, our model calculations clearly indicate that the anodic peak of the chemical capacitance is caused by highly compressed oxygen in closed pores corresponding to gas pressures of ∼10^4^ bar.

## 2 Experimental Section

### 2.1 Sample Preparation

Yttria-stabilized zirconia (YSZ) single crystals (5 × 5 × 0.5 mm^3^, (100)-oriented, 9.5 mol % Y_2_O_3_; CrysTec, Germany) were used as electrolyte substrates. For the fabrication of the working and the counter electrodes, LSC thin films were deposited on the YSZ single crystals using pulsed laser deposition (PLD). The corresponding target was prepared via Pecchini synthesis followed by a calcination of the obtained powder for 2 h at 1000 °C. Afterward the powder was pressed to a pellet by cold isostatic pressing (300–310 MPa) and subsequently sintered in air for 12 h at 1200 °C.

**Figure 1:**
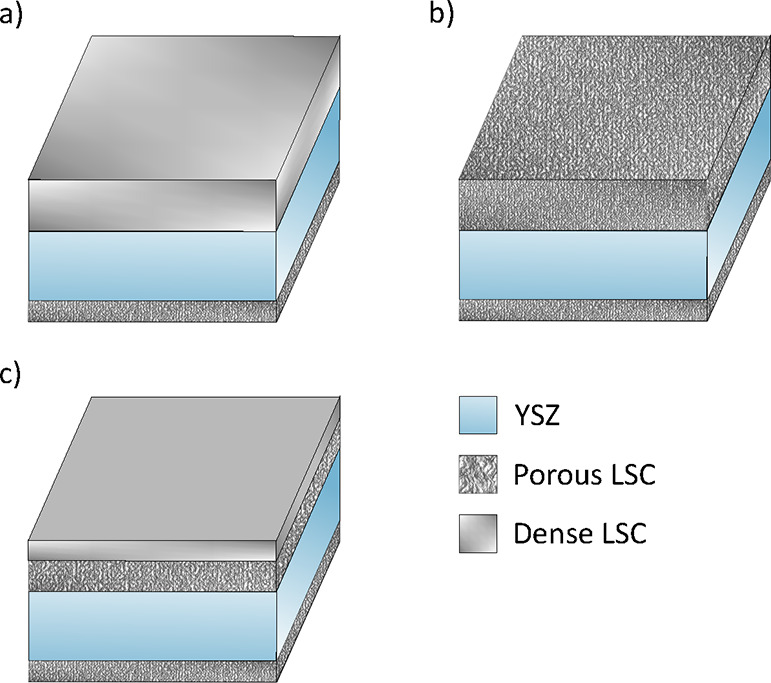
Sketches of the different sample types investigated in this study: dense (a), porous (b), and porous/capped (c).

Ablation of the target was done in a vacuum chamber using a KrF excimer laser (Complex Pro 201F, Coherent LaserSystems GmbH & Co. KG, Germany) with a wavelength of 248 nm. In the first step, porous LSC counter electrodes were deposited at an oxygen partial pressure of 0.4 mbar and at a substrate temperature of 450 °C. Earlier studies revealed that electrodes prepared with these parameters exhibit very low polarization resistances because of an increased inner surface area (open porosity).^40,41^ Three sample types were then prepared, which differ in microstructure (i.e., porosity and surface area) of the working electrode: (a) polycrystalline dense, (b) porous, and (c) porous films with a dense capping layer on top (denoted as porous/capped) (see Figure 1).

Table 1 displays the deposition parameters for the three different sample types. The deposition temperatures were measured with a pyrometer which was adjusted to the emissivity of YSZ. For the deposition of dense films, the laser energy was adjusted such that the fluence inside the vacuum chamber was approximately 1.1 J/cm^2^. For porous films of similar composition, the laser energy was increased prior to the deposition yielding a fluence of about 1.4 J cm^2^.

**Table 1:** Deposition Parameters for the Five Different Sample Types Investigated in This Study

sample type	temperature (°C)	oxygen partial pressure (mbar)	target–substrate distance (cm)
dense	600	0.04	6
porous	450	0.4	5
porous/capped	450/600	0.4/0.04	5/6

For all different sample types, the laser was operated with a pulse repetition rate of 5 Hz. By varying the pulse number, total electrode thicknesses between 40 and 100 nm were obtained (slightly thicker films were used for transmission electron microscopy (TEM) measurements). The film thicknesses were determined using a profilometer (DektakXT, Bruker, U.S.A.). The porous part of the porous/capped films accounted for about 80% of the total electrode thickness. Immediately after the deposition of this porous part, the parameters were changed to those for the dense films in order to deposit a dense capping layer with a thickness in the range of 10 to 20 nm. The deposition parameters of the dense films were chosen on the basis of earlier studies, which confirmed dense packing of columnar grains by TEM cross sections.^42,43^ The parameters for the porous films were also taken from a previous study, where the porosity of the respective films was confirmed via TEM bright field cross sections and high-angle annular dark field (HAADF) measurements.^40^ After the deposition, all samples were cooled with a rate of 15 °C per minute. The compositions of the films were analyzed by dissolving them in hydrochloric acid and using inductively coupled plasma-mass spectroscopy (ICP-MS). This revealed an average film composition for all sample types of La_0.599±0.019_Sr_0.411±0.010_Co_0.990±0.016_O_3−δ_.

After the PLD process, microstructuring of the working electrodes was done via photolithography and ion beam etching. For the photolithography process, the samples were coated with 4 × 100 μL of photoresist (ma-N 1420 MicroResist Technology, Germany) using a spin-coater, spinning the samples for 1 min every 100 μL. After heating the samples for 5 min at 100 °C in order to evaporate the excess solvent, they were exposed to UV light (350 W, USHIO 350DP Hg, Ushio, Japan) for 1 min through a patterned shadow mask to obtain circular microelectrodes with a diameter of 250 μm. The nonilluminated parts of the photoresist were removed with a developer solution (ma-D 533/s, MicroResist Technology, Germany). These areas of the LSC films were then removed via ion beam etching (KDC 40, Kaufman & Robinson Inc., U.S.A.) using a diffuse Ar plasma operated at 9 × 10^–4^ mbar Ar with a beam voltage of 500 V and a beam current of 10 mA. Finally, the remaining photoresist was carefully removed with a clean room wipe, which was soaked in ethanol.

### 2.2 Impedance Spectroscopy

Measurements were performed by placing the samples in a closed fused silica apparatus and heating them in a tube furnace to temperatures between 460 and 608 °C. The temperature was measured with a type S thermocouple, which was positioned within 1 cm distance to the sample. Electrical contact of the counter electrodes was realized by placing the samples on a platinum mesh. The (working) microelectrodes were contacted by means of platinum–rhodium needles using a microscope camera. Impedance measurements with DC bias voltages from 0 to 440 mV were carried out using an Alpha-A High Performance Frequency Analyzer with an Electrochemical Test Station POT/GAL 30 V/2 A (both: Novocontrol Technologies GmbH & Co. KG, Germany). An alternating root-mean-square voltage of 10 mV was employed, and impedance spectra were measured in the frequency range of 10^6^ to 10^–2^ Hz with 5 data points per decade. DC voltages and currents were also measured with the Electrochemical Test Station. All measurements were performed in synthetic air (99.999%, Messer Austria GmbH, Austria).

### 2.3 X-ray Diffraction

The crystal structure of differently prepared LSC thin films was analyzed via X-ray diffraction (XRD) with a Cu radiation source in grazing incidence geometry using an Empyrean X-ray diffractometer (Malvern Panalytical, U.K.). A parallel beam mirror on the incident beam side and a parallel plate collimator together with a scintillation detector on the diffracted beam side were used for the scans, which were performed at an incidence angle of 2°.

### 2.4 Inductively Coupled Plasma Mass Spectrometry

To determine the elemental composition of LSC thin films, an inductively coupled plasma mass spectrometer (ICP-MS) equipped with a quadrupole mass filter and a collision cell (iCAP QC, ThermoFisher Scientific, Germany) was used. Prior to the analysis, a two-step dissolution process was applied according to a previous study^43^: In a first step, the water-soluble species of Sr possibly formed on the surface of pristine LSC samples were dissolved in 5 mL freshly prepared ultrapure water (BarnsteadTM EasypureTM II, 18.2 MΩcm) for 30 min. In a second step, the remaining LSC thin film was completely dissolved with 100 μL of concentrated HCl. The obtained data was processed using Qtegra software (ThermoFisher Scientific, U.S.A.). To minimize the influence of polyatomic interferences, the kinetic energy discrimination (KED) mode was used. Herein undesirable molecule ions are suppressed in the collision cell containing a mixture of helium with 7% hydrogen. Observed signal intensities were normalized using the signal response for the internal standard and finally converted into concentration units by means of external aqueous calibration. Derived Cu signals were constant over each measurement session (less than 5% relative standard deviation for the whole measurement period, indicating the absence of temporal trends), and no significant difference in Cu-response between samples and calibration standards was observed.

### 2.5 Transmission Electron Microscopy

Electron-transparent lamellae of about 10 μm length were prepared from additional films of each sample type via standard lift-out techniques with a focused ion beam/scanning electron microscopy (FIB/SEM) system (Scios 2 DualBeam, Thermo Fisher Scientific, Germany), operating with a Ga-ion beam at 30 kV accelerating voltage. Final low-voltage cleaning of the lamellae was conducted at 5 and 2 kV. Bright field transmission electron microscopy (BF-TEM) was performed on a 200 kV FEI TECNAI F20.

## 3 Results

### 3.1 Film Characterization

**Figure 2:**
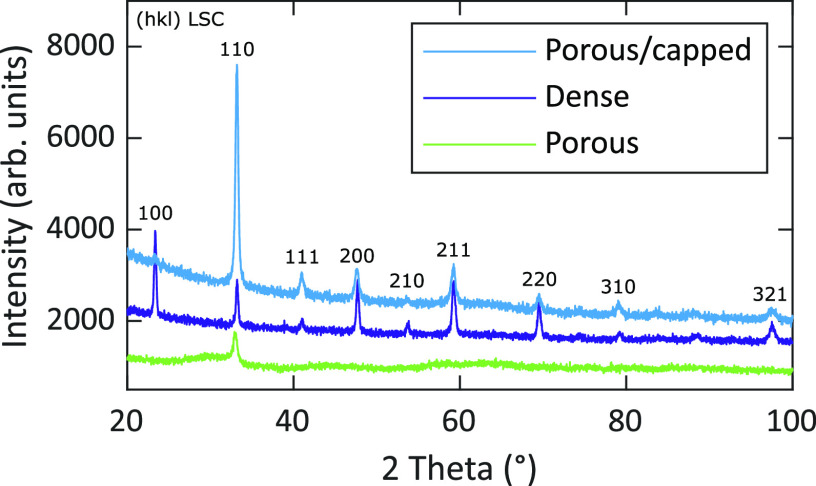
XRD diffractograms of dense, porous, and porous/capped films measured in the grazing incidence geometry.

XRD measurements revealed the same crystal structure for LSC films of all different sample types, and all reflexes could be assigned to the LSC perovskite phase. In Figure 2, all LSC peaks in the different diffractograms were labeled according to the pseudocubic structure. For the porous LSC film, only one distinct peak is visible, which indicates a low degree of crystallinity.

In order to investigate the open porosity (i.e., the surface area) of the three different sample types, the surface compositions of the LSC thin films were analyzed via ICP-MS measurements in accordance with the approach described in previous works.^40,43,44^ Former studies reported a water-soluble Sr surface species being present at the surface after the preparation of the thin films.^40,43,45^ Thus, by analyzing the amount of this species, it is possible to compare the surface area of the different thin films. At first, leaching was done with pure H_2_O, particularly dissolving surface species, followed by a subsequent chemical analysis via ICP-MS. Then the films were completely dissolved in hydrochloric acid and an ICP-MS measurement was again conducted to examine the amount of Sr in the bulk (*c*_bulk_). In order to compare the data obtained for the three different sample types, the amount of water-soluble Sr (*c*_surf_) was related to the total amount of Sr in the film (*c*_total_ = *c*_surf_ + *c*_bulk_). The corresponding results are shown in Table 2.

**Table 2:** Ratio of Water-Soluble Surface Sr (*c*_surf_) to Total Sr (*c*_total_ = *c*_surf_ + *c*_bulk_) of LSC Thin Films

sample type	*c*_surf_/*c*_total_ (%)
dense	0.86
porous	6.75
porous/capped	1.09

We assume that the water-soluble Sr species is homogeneously distributed across the surface of all different films. A higher amount of dissolved Sr thus suggests a larger accessible surface area. The poly/dense and the porous/capped films have similar amounts of water-soluble surface Sr, which indicates that the top layer of the porous/capped films has a similar morphology as the dense films. Moreover, we can conclude that the majority of the initially open pores in the bottom layer of the porous/capped films becomes closed because of the deposition of a dense film on top. The amount of water-soluble Sr of the porous films is much higher than for the dense and the porous/capped samples. In accordance with a former study,^40^ we therefore assume open porosity for the porous films leading to a surface area that is 7 to 8 times larger compared with the dense films.

**Figure 3:**
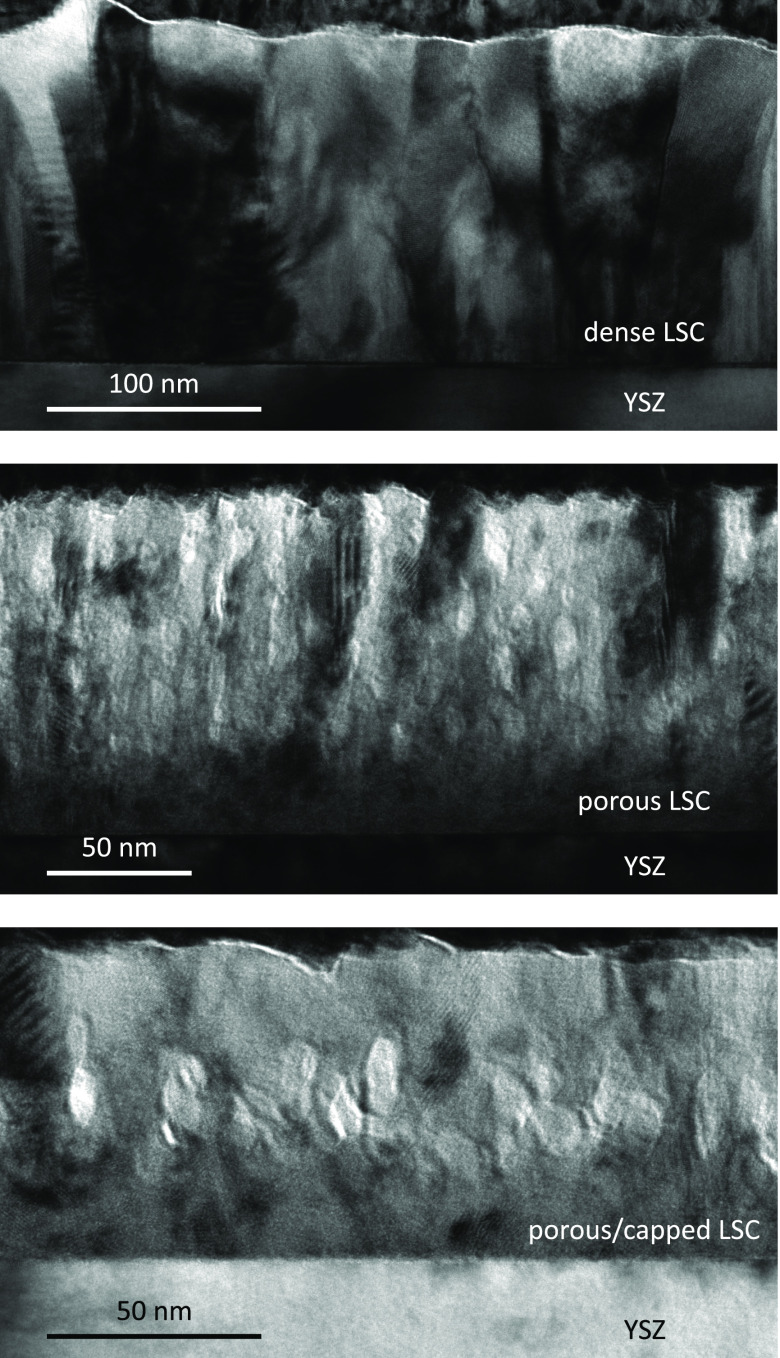
BF-TEM cross sections of dense, porous, and porous/capped LSC films.

BF-TEM measurements were performed to further analyze the porosity and nanostructure of the different LSC thin films (see Figure 3). TEM cross sections of a dense film reveal dense packed and columnar growth in accordance with an earlier study,^42^ where the same oxygen partial pressure and substrate temperature was used during deposition.

Similar to a previous work on LSC,^40^ porous films exhibit a rather dense growth in the first approximately 25 nm followed by a porous nanostructure confirming the findings from the ICP-MS measurements. A similar nanostructure is found for the porous/capped film; however, above the porous layer, the film looks dense. This is again in accordance with the conclusion from the ICP-MS analysis suggesting that the pores get closed because of the deposition of a dense capping layer on top.

### 3.2 Impedance Spectroscopy

**Figure 4:**
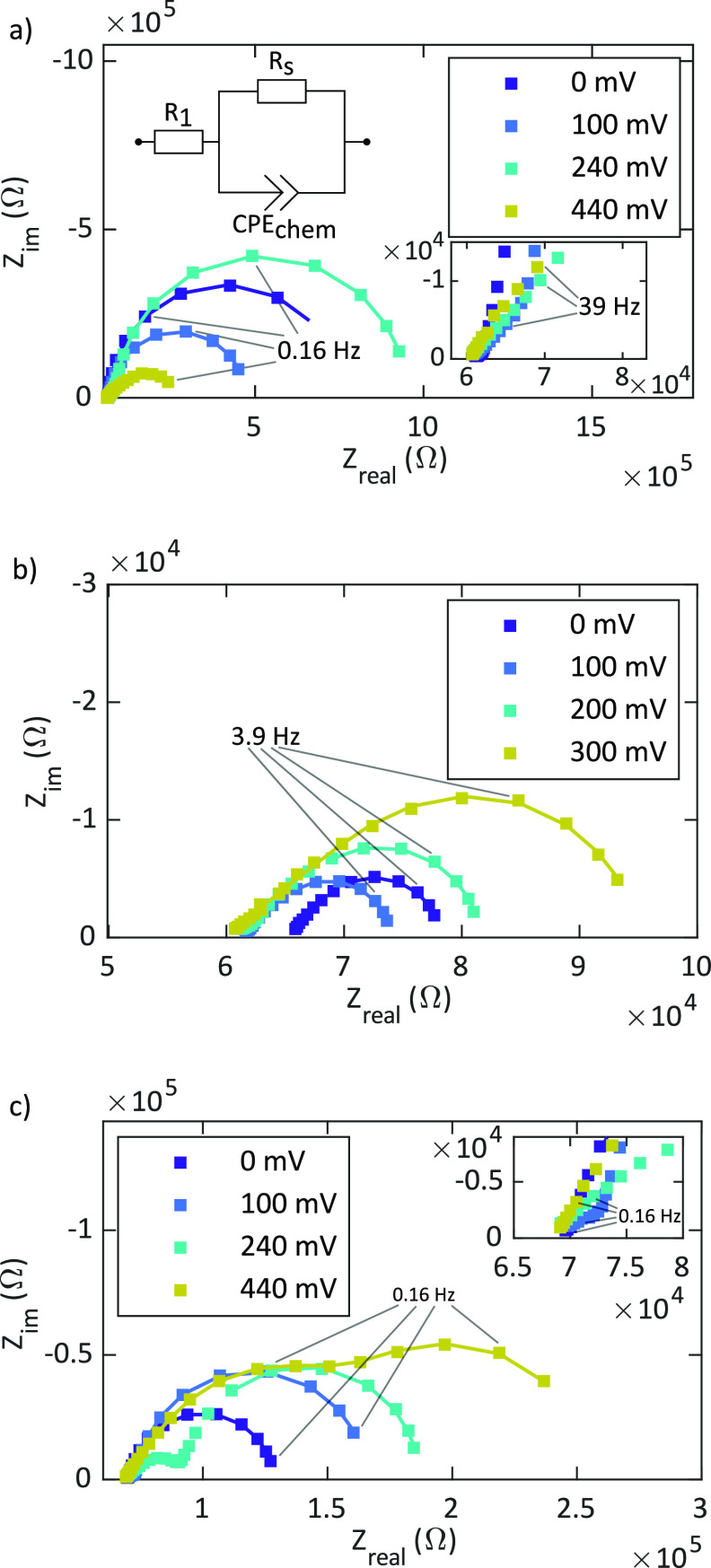
Impedance spectra of a dense (a), a porous (b), and a porous/capped (c) LSC thin film microelectrode at various anodic DC bias voltages (*U*_DC_) measured at 460 °C. Lines represent fits according to the sketched equivalent circuit in (a) (with the exception of spectra at 440 mV in (a) and (c) and at 300 mV in (b), where an additional R/CPE element was used).

Figure 4 shows exemplary impedance spectra of the different thin film microelectrodes used in this study at various anodic DC bias voltages.

The four spectra displayed in each plot are examples of measurement cycles with bias voltages from 0 to 440 mV and steps of 20 mV. All impedance spectra contain a high frequency *x*-axis intercept which corresponds to the ionic transport resistance of the YSZ electrolyte (*R*_YSZ_), in accordance with the literature.^37,40,46^ This electrolyte resistance is temperature dependent^46^ but shows no dependence on the applied bias voltage. *R*_YSZ_ was determined by extrapolating the electrode-related impedance to the *x*-axis, and this value was also used for subtracting the ohmic overpotential from the applied bias (see the section Chemical Capacitance Analysis). The slightly different electrolyte resistance for the spectrum at open circuit conditions in Figure 4b compared with the spectra under DC bias voltages of the porous electrode reflects a temperature difference of about 2 °C. In addition, a semicircular feature at low frequencies is obtained for all spectra. The size of this feature varies depending on the applied DC bias. In agreement with former studies, this low-frequency arc is attributed to the oxygen surface exchange resistance *R*_s_ and the chemical capacitance *C*_chem_ of the LSC working electrode.^36,37,40,41,43,47^ Please note that the oxygen exchange kinetics at open circuit conditions is much faster than for similar bias-dependent measurements for LSCF reported in literature^37^ (*R*_s_ = 50 Ω cm^2^ at a significantly higher temperature of 700 °C^37^), most probably because of the different preparation parameters and thermal history of the films. It is therefore not surprising that the bias dependence of *R*_s_ is also different for the measurements of this study. Moreover, the oxygen exchange rates are strongly affected by defect concentrations (holes, oxygen vacancies)^48,49^ and those vary with the applied anodic bias. Finally, also some surface changes at high voltages may occur.^50^ Thus, different and also complicated bias dependencies of the differential resistance *R*_s_ may result (e.g., the surprising increase and decrease of *R*_s_ for the dense film in Figure 4a). However, a more detailed analysis of the oxygen surface exchange resistance is beyond the scope of this paper.

Furthermore, additional electrode-related features can be observed at intermediate frequencies. As can be seen in Figure 4, those differ between sample types and show a dependency on the applied DC voltage. The shape of these intermediate frequency features varies between arc-like and Warburg-like slopes, though a finite Warburg element alone could never fit the spectra properly. Such intermediate frequency contributions are known from literature data on LSC electrodes^40,42,43^ and are usually attributed to processes which are located at the YSZ/LSC interface. The Warburg-like behavior found here in some cases may also indicate the onset of an oxygen diffusion limitation in LSC. In general, intermediate frequency contributions become larger with increasing DC voltage. Since the focus of this study is on the analysis of the chemical capacitance *C*_chem_, we did not further investigate those intermediate frequency features.

*C*_chem_ was evaluated as long as the low frequency semicircle was clearly separated and accounted for a major part of the respective spectrum (i.e., when the polarization resistance of the electrode was determined by the surface exchange reaction). Then, one can assume that a major part of the electrode overpotential is transferred to an oxygen chemical potential change in the electrode.^35,36^ When the intermediate and the low-frequency contributions were similar in size (e.g., spectra at 440 mV in Figure 4a,c and at 300 mV in Figure 4b), the determination of *C*_chem_ and the corresponding chemical potential from the nominal electrode overpotential (see below) became somewhat doubtful. Therefore, such data were excluded from the following chemical capacitance analysis and a different equivalent circuit was used to describe these spectra (two R/CPE elements and a resistance in series). However, the majority of the obtained spectra allow a reasonable analysis of *C*_chem_ since the resistance associated with the intermediate frequency feature is considerably smaller than *R*_s_ as can be seen in Figure 4. The low-frequency feature of these spectra was described by a parallel connection of a constant phase element (CPE_chem_) and a resistance (*R*_s_). A constant phase element with the impedance
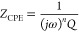
3considers the nonideal behavior of a capacitance. The CPE parameter *Q* and the exponent *n*, which quantifies the deviation from an ideal capacitance, were both obtained from nonlinear least-squares fitting and used to calculate the corresponding capacitance via^51^
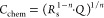
4

An additional serial resistance in the equivalent circuit (*R*_1_) considers the above-mentioned contributions from the electrolyte (*R*_YSZ_) and the intermediate frequency feature (see circuit in Figure 4a). Fitting of the spectra to one R/CPE element, considering the low frequency semicircle, and a serial resistance *R*_1_ allowed the most reliable determination of the chemical capacitance, which was the focal point of this paper. Tables S1–S3 show exemplary fitting results for each sample type according to this equivalent circuit.

### 3.3 Chemical Capacitance Analysis

As shown in a previous study,^40^ the porous counter electrodes exhibit very low polarization resistances. Moreover, they have an active area being at least 500 times larger than that of the microelectrodes. Thus, their influence on the measured impedances is negligible and the overpotential of the (working) microelectrodes η_WE_ is determined as follows
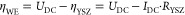
5where *U*_DC_ stands for the applied DC bias voltage, *I*_DC_ is the DC current, and η_YSZ_ denotes the ohmic overpotential caused by the finite ionic conductivity of the electrolyte.

**Figure 5:**
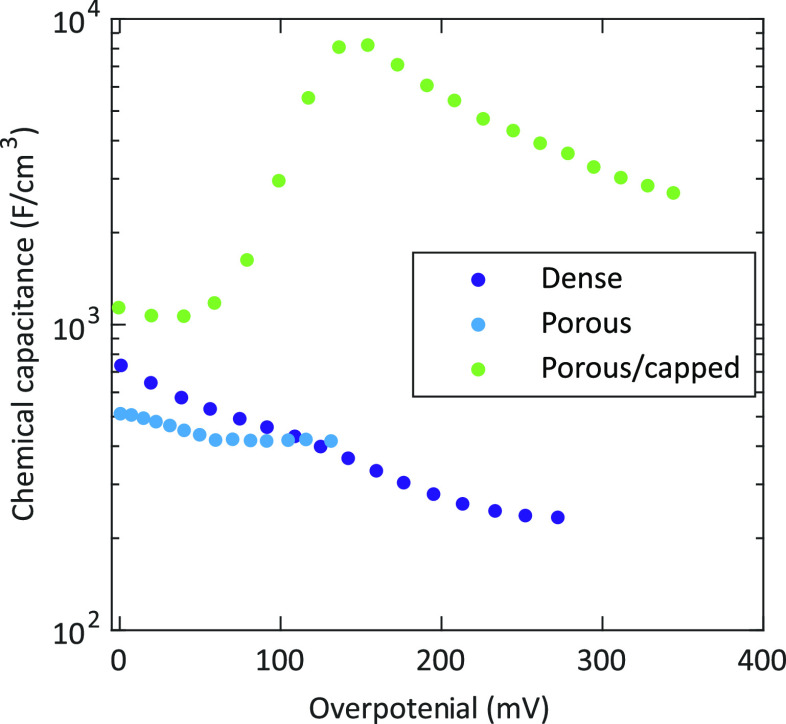
Chemical capacitance of a dense, a porous, and a porous/capped thin film microelectrode as a function of the electrode overpotential, measured at 460 °C.

Figure 5 shows the chemical capacitances of the three different sample types, measured at 460 °C in synthetic air. All values were normalized to the respective electrode volume, without correcting for the pore volume of porous and porous/capped electrodes. In the case of porous electrodes, the chemical capacitance could only be evaluated up to η_WE_ ≈ 130 mV because of a strong merging of the intermediate and the low frequency feature.

While the curves for the dense and the porous films are somewhat similar, a very different and completely unexpected behavior is found for porous/capped electrodes: a capacitance peak with extremely high values. The latter is in the main focus of our study; however, we first discuss the ”standard behavior” of the other films. The chemical capacitances of these films decrease with increasing overpotential and level off at about 250 and 60 mV, respectively. Such a behavior is qualitatively consistent with previous studies on LSC and similar mixed conducting oxides upon anodic polarization.^35−37^ The corresponding chemical capacitance is caused by the redox reaction of the transition metal cations and its dependence on the oxygen chemical potential. This reaction is accompanied by a change in concentration of oxygen vacancies, which are the minority charge carriers under these conditions. The general behavior of a decreasing chemical capacitance with increasing overpotential can thus be attributed to the decrease of the oxygen vacancy concentration with increasing chemical potential of oxygen.^52−57^ As shown in the literature,^35,36^ the concentration of oxygen vacancies and other defects solely depend on the chemical potential of oxygen in the electrode  according to
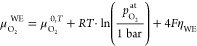
6regardless of the respective contribution of the overpotential η_WE_ and the actual atmospheric partial pressure  (assuming an ideal gas). Here,  stands for the chemical potential of oxygen at 1 bar and *R*, *T* are the usual notations of the universal gas constant and the temperature, respectively. Hence, applying anodic (i.e., positive) overpotential leads to an increase of the chemical potential of oxygen. The validity of eq 6 requires that the transport of charge carriers in the thin film is fast compared to the oxygen surface exchange reaction. In addition, we neglect that a certain part of the electrode overpotential η_WE_ refers to the intermediate frequency feature and does not change  in the entire electrode. However, as long as the low-frequency arc is the dominant feature in the corresponding impedance spectra (see above), eq 6 is a reasonable approximation.

Moreover, one may introduce a nominal oxygen partial pressure inside the working electrode , as similarly done in former studies,^35,36^ according to
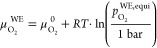
7and thus we get
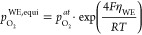
8which further illustrates the relation between oxygen partial pressure, the electrode’s overpotential, and the oxygen chemical potential. However, please note that considering the high anodic overpotentials of the measurements in this study, eqs 7 and 8 exceed their range of validity, since ideal gas behavior is assumed. The extension to a real gas is discussed in more detail below.

The overall lower capacitance values for the porous films may be a result of the oxygen exchange reaction taking place along the open pores, which can lead to inactive parts of the film. Apart from not considering the porosity of these films for the volume normalization, this effect contributes to a further reduction of the effective volume. This explanation is also supported by the higher capacitance values of the porous/capped electrodes at low overpotentials. The different slopes of the dense and the porous electrodes may be attributed to strain effects and/or contributions from grain boundaries since porous films show a low degree of crystallinity (see Figure 2). A more detailed analysis of the decreasing chemical capacitance is beyond the scope of this study.

**Figure 6:**
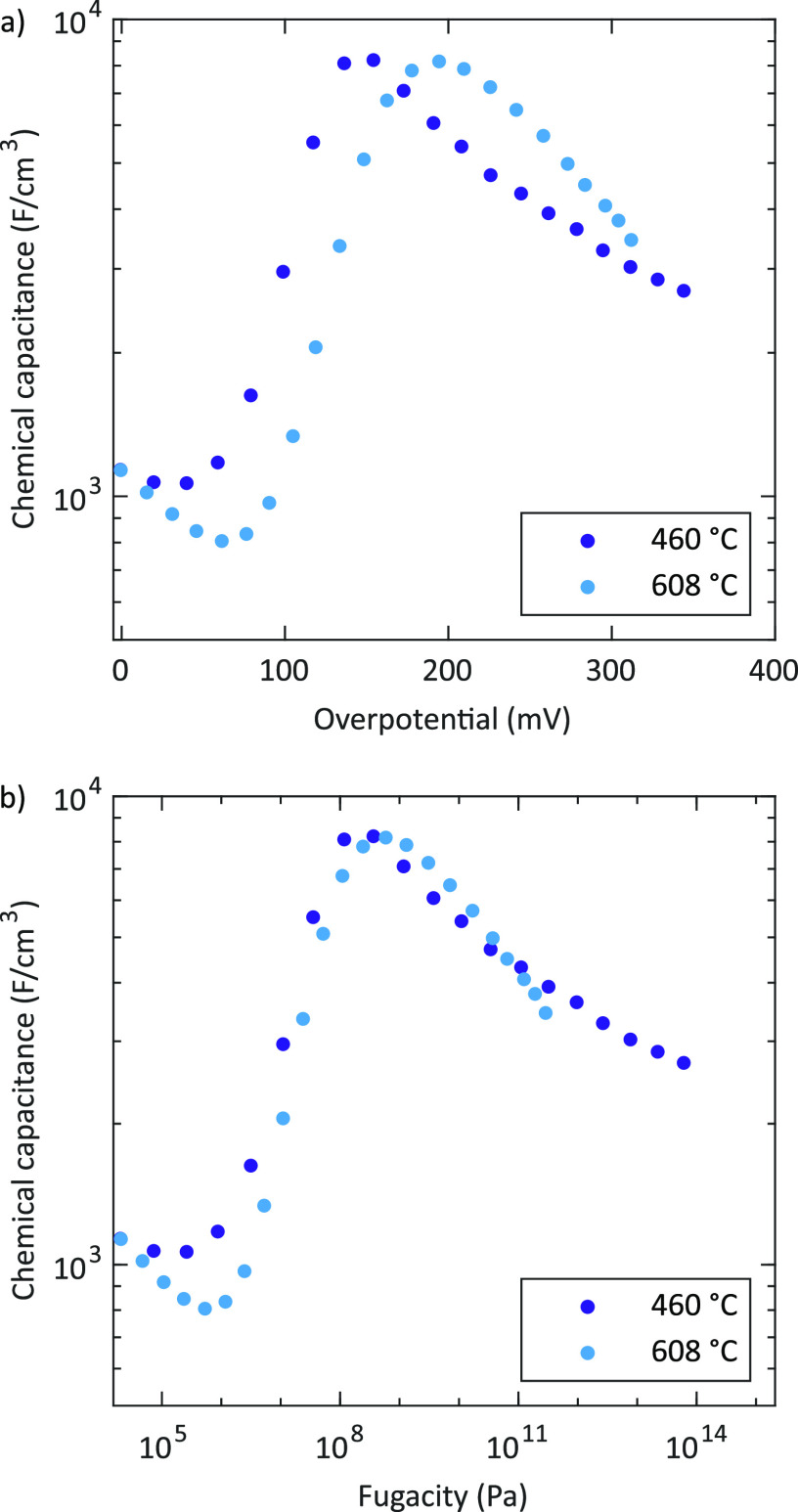
Chemical capacitance of porous/capped thin film microelectrodes measured at 460 and 608 °C as a function of the electrodes’ overpotential (a) and equivalent fugacity (b). Two different samples were used for the different temperatures.

The chemical capacitance of porous/capped electrodes also decreases with increasing overpotential, however only up to about 40 mV. At higher overpotentials, these electrodes exhibit a pronounced capacitance peak at about 150 mV with maximum values up to 8200 F/cm^3^. Thus, they display a completely different and unexpected behavior. After reaching this maximum, the chemical capacitance again decreases with increasing overpotentials. This capacitive peak was found for all porous/capped microelectrodes on several different samples at overpotentials of about 150 and 190 mV for temperatures of 460 and 608 °C, respectively (see Figure 6a). Interestingly, the chemical capacitances for different temperatures show a considerable overlap when plotting against the electrodes’ oxygen fugacity (see discussion below) as depicted in Figure 6b. Additionally, the peak remains with only little changes if electrodes were cycled stepwise up to about 300 mV and subsequently back to 0 mV. To the best of the authors’ knowledge, such an increase of the chemical capacitance under anodic polarization in synthetic air has not been reported in the literature yet.

This behavior cannot be explained by the standard defect chemical interpretation of the chemical capacitance^35,36^ and clearly suggests the involvement of an additional species in the redox reaction contributing to the chemical capacitance. Since this phenomenon was only observed for the porous/capped samples, we may conclude that closed porosity is required for the occurrence of this chemical capacitance peak.

In the following discussion, we show that the appearance of this pronounced capacitance maximum, its temperature dependence and overpotential dependence, as well as its absolute value can be excellently explained by high pressure oxygen storage in closed pores, provided that real gas equations instead of ideal gas laws are considered.

## 4 Mechanistic Discussion

**Figure 7:**
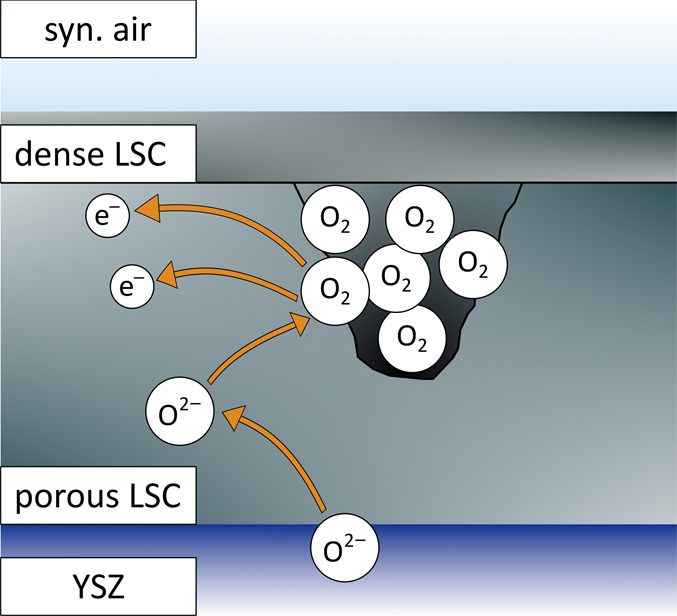
Sketch of neutral oxygen gas formation and storage in closed pores of a porous/capped film.

A mixed ionic and electronic conducting oxide has the ability to shift its nonstoichiometry (i.e., to change δ of La_0.6_Sr_0.4_CoO_3−δ_, upon external drivers such as oxygen partial pressure, temperature, or bias voltage). However, as already discussed above, this phenomenon can only explain the baseline of the chemical capacitance curve with decreasing *C*_chem_ values. Therefore, another redox process has to contribute to the chemical capacitance. On the basis of the requirement of closed porosity in order to obtain the capacitive peak at anodic overpotentials, we now consider the formation and storage of neutral oxygen gas in closed pores as an additional contribution to *C*_chem_. Figure 7 depicts this reaction in closed pores of a porous/capped film.

**Figure 8:**
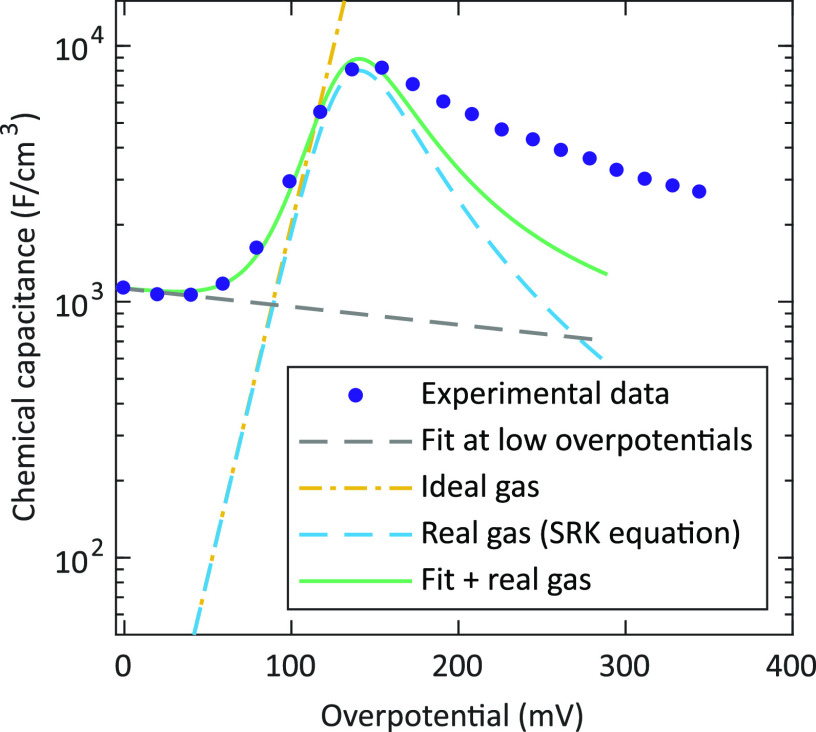
Calculated chemical capacitance of an ideal  and a real  high-pressure oxygen gas and experimentally obtained chemical capacitance of a porous/capped electrode at 460 °C as a function of the electrode overpotential. The gray dashed line represents the fit and the corresponding extrapolation of the experimental capacitance values at low overpotentials . Additionally, the green curve shows the sum of this extrapolation and the capacitance of the real gas approach.

Assuming ideal gas behavior, the corresponding O_2_ gas reservoir acts as a chemical capacitor with a capacitance according to
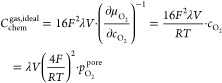
9with the film porosity λ and the oxygen partial pressure in closed pores . This relation yields a continuous increase of the capacitance with increasing partial pressure or correspondingly, with increasing anodic overpotential (see Figure 8). Even though a strong increase was indeed found above 40 –60 mV, the measured capacitance curve is not predicted by eq 9. Rather, the capacitance decreased after reaching a maximum value which appeared at overpotentials at about 150 and 190 mV for temperatures of 460 and 608 °C, respectively. According to eq 8, these overpotentials correspond to high equivalent partial pressures between 1.5 × 10^3^ and 8 × 10^3^ bar, respectively, and thus to values beyond the limits of ideal gas behavior. This is even more true for overpotentials far beyond the maximum; 300 mV at 460 °C, for example, results in a nominal pressure of 3.7  ×  10^7^ bar.

Consequently, we have to consider real gas behavior. In the following, we do this in terms of the Soave–Redlich–Kwong (SRK) equation of state^58^ according to
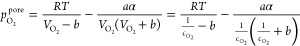
10
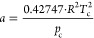
11
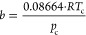
12
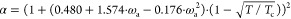
13with  denoting the molar volume of O_2_ and *T*_c_ = 154.6 K and *p*_c_ = 50.46 bar being the critical temperature and critical pressure of O_2_,^59^ respectively. The α parameter contains the acentric factor of oxygen ω_a_ = 0.022, which takes account of the influence of intermolecular forces depending on the orientation of the molecule.^59^ The fugacity coefficient ϕ corresponding to the Soave–Redlich–Kwong (SRK) equation of state can be calculated as follows^58^
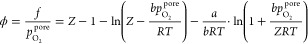
14with the fugacity *f* and the compressibility factor *Z* being
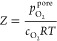
15

For the calculation of the chemical capacitance of a real gas, it is necessary to consider the fugacity coefficient for the oxygen chemical potential according to
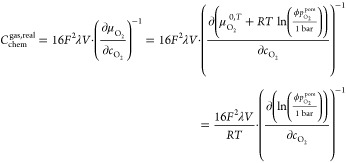
16

By taking the values for  and ϕ calculated according to eqs 10 and 14, respectively,  in eq 16 was determined numerically.

In order to compare the capacitance obtained from this calculation with our experimental data, the overpotential η_WE_ relative to the oxygen partial pressure in synthetic air is related to the fugacity *f* and the fugacity coefficient ϕ by
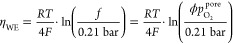
17

As a consequence, for high anodic overpotentials, the equivalent oxygen partial pressure  defined in eq 8 should be replaced by the equivalent oxygen fugacity  as follows
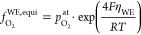
18

Figure 8 shows the calculated chemical capacitance of an ideal and a real oxygen gas as well as the experimental values of a porous/capped electrode for a temperature of 460 °C. The overall closed porosity of the porous/capped film (including the dense capping layer) of the calculated curves was the only free parameter in the calculation and was adjusted in order to obtain the best agreement with the experimental data. This yielded a closed porosity of λ = 0.0425. The chemical capacitance of a real gas thus exhibits an increase with a maximum at a very similar overpotential as the experimentally determined capacitance. Moreover, unlike for the ideal gas approach, the capacitance of the real gas decreases with increasing overpotential, that is, with increasing oxygen concentration, after reaching a maximum at 140.5 mV. The sum of the calculated real gas capacitance and the extrapolation of the defect-related LSC chemical capacitance at low overpotentials agrees rather well with the experimentally obtained capacitance of a porous/capped electrode.

At high overpotentials, the calculated capacitances are lower than the experimental values. This deviation may have several possible reasons including a continuous loss of oxygen e.g. via leaky grain boundaries in the dense top layer or some errors in determining the proper local fugacity due to neglecting any interfacial and/or transport overpotentials. Additionally, the high oxygen pressure may induce redox processes in or near pores which could further contribute to the increased experimental capacitances at high overpotentials, for example, SrO_2_ formation from SrO and O_2_. From thermodynamic data,^60^ the stability limit for the reaction of SrO to SrO_2_ would correspond to an overpotential of 67 mV at 445 °C, which is lower than the overpotentials for which deviations from our model are observed. However, for the system in our study, it needs to be considered that the thermodynamics of SrO on or in LSC are different than for bulk SrO. We therefore expect a shift of the corresponding stability limit to higher overpotentials at which the reaction could also contribute to the increased chemical capacitance. Further deviations may occur because of particle interactions at extremely high densities.

However, because of the very good agreement of the calculated curve with the experimental data at moderate overpotentials and the fact that the suggested mechanism explains the decrease of the capacitance with increasing overpotential after reaching a maximum, we conclude that the formation of high-pressure oxygen gas in closed pores is responsible for the strong capacitance increase upon anodic polarization. Furthermore, calculations based on the presented real gas model yield a shift of the capacitance maximum to higher overpotentials with increasing temperature as observed in our experiments. The calculations based on the real gas model predict a peak shift from 141 mV at 460 °C to 174 mV at 608 °C and the corresponding experiments yield peaks at 154 mV and 194 mV, respectively. As already described above, the chemical capacitance curves measured at 460 and 608 °C overlap when they are plotted as a function of the electrodes’ oxygen fugacity according to eq 18. This overlap indicates that the storage of highly pressurized O_2_, which causes the *C*_chem_ increase, occurs at particular fugacity values.

As already mentioned above, the capacitance peak was reproducibly found when performing several measurements on one electrode. Besides, no morphological changes were visible in the optical microscope after measurements on porous/capped electrodes at overpotentials >300 mV. This indicates that closed pores were largely not destructed (i.e., opened) in our measurements despite (mechanical) gas pressure values in the range of 10^4^ bar. Nevertheless, the long-term stability of such closed pores upon polarization can still be a serious issue in real SOEC electrodes. Tiny closed pores at interfaces or within electrode particles may be exposed to extremely high true (mechanical) gas pressures of ∼10^4^ bar for long times which may cause degradation (e.g., delamination or pore and crack formation).

The existence of this capacitance peak due to closed porosity may also be used as a nondestructive observation tool during cell operation for detecting the presence of closed pores at an early stage in a real SOEC anode. Even if the ratio between closed pore volume to LSC bulk is a factor of 300 lower than in our model electrodes, a shoulder is visible on the defect-related *C*_chem_ baseline. Moreover, the presented electrochemical method is capable of detecting any high pressure oxygen build-up in closed porosity at the anode side of SOECs, regardless of the location, as the internal oxygen formation is always associated with an increased chemical capacitance. Thus, it should be possible to detect closed pores or cracks (and the development of high pressure oxygen therein) at the anode/electrolyte interface and even in the electrolyte close to that interface. Nevertheless, the kinetics of the associated oxygen exchange reaction is important since 1/(*R*·*C*_chem_) determines the frequency of the respective feature in the impedance spectrum (*R* being the resistance of the oxygen production). Therefore, a large exchange resistance *R* and slow electron transport in the electrolyte might lead to extremely low frequencies of <10^–3^ Hz, which may render impedance measurements difficult.

**Figure 9:**
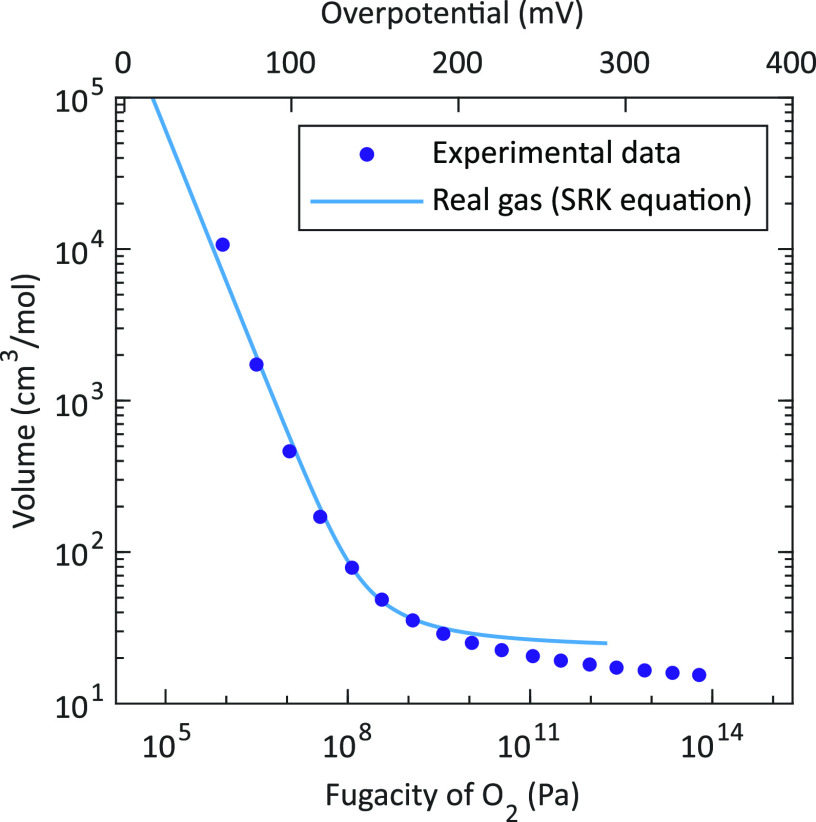
Molar volume according to the real gas model shown in eq 10 compared to the molar volume *V*_m_ calculated from experimental chemical capacitance data shown in Figure 8 as a function of the electrode overpotential and the corresponding fugacity.

In addition to the implications of this effect for the oxygen electrode of SOECs, we can also attempt to extract fundamental data of pressurized O_2_ at high temperatures that are otherwise hardly accessible. Figure 9 displays the oxygen molar volume calculated from a cumulative numerical integration over the peak of the experimental capacitance data shown in Figure 8. Subtracting the extrapolation of the fit at low overpotentials  according to
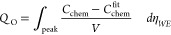
19yields the volume specific charge *Q*_O_ of the oxygen gas formation. The corresponding molar volume  was calculated as follows:
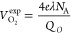
20with the Avogadro constant *N*_A_. The oxygen molar volume  obtained from integrating the curve of the real gas model in Figure 8 is also displayed. Both curves demonstrate that we may face oxygen molar volumes in the 30 cm^3^/mol range. For the purpose of comparison it is worth mentioning that solid oxygen exhibits about 15 cm^3^/mol at 10 GPa and room temperature.^61^

**Figure 10:**
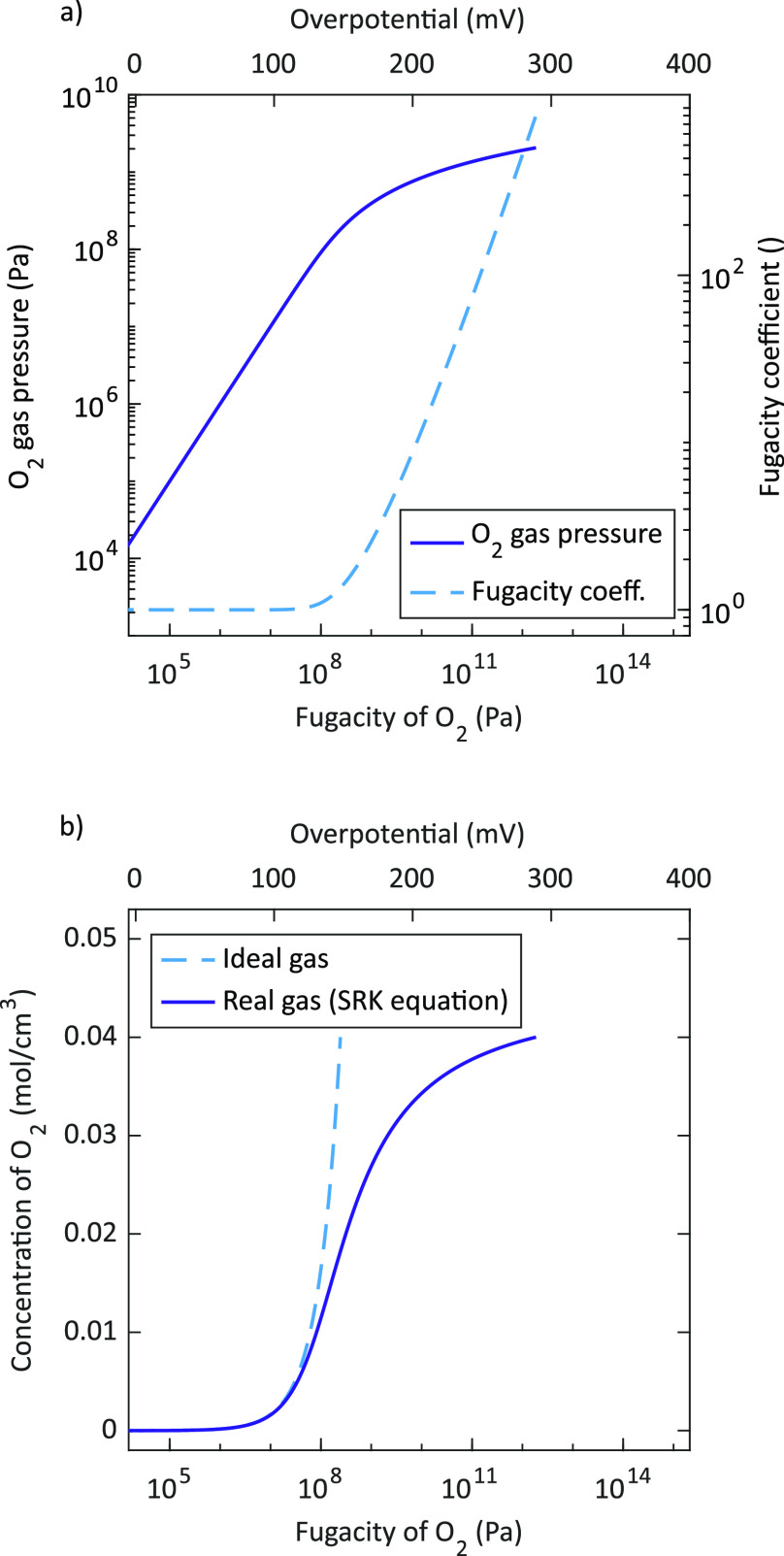
(a): Oxygen gas pressure  and fugacity coefficient ϕ obtained from eqs 10 and 14, respectively. (b): Oxygen concentration  according to the ideal and real gas model, respectively. All quantities are plotted as a function of the fugacity and the corresponding electrode overpotential at 460 °C.

Figure 10a illustrates the deviation of the gas pressure according to the real gas model from the equivalent partial pressure calculated via Nernst’s equation as shown in eq 8. Here it is clearly demonstrated that at overpotentials higher than approximately 140 mV (equivalent to a fugacity of about 148 MPa) the oxygen gas pressure does not increase exponentially with increasing overpotential (or linearly with the fugacity). In particular, the capacitance maximum obtained for the porous/capped electrode at 460 °C corresponds to a (mechanical) gas pressure of about 2000 bar and thus already deviates from the equivalent partial pressure  according to Nernst’s equation as defined in eq 8 which yields 2800 bar and is actually the fugacity *f* of oxygen (see eq 18). The difference between gas pressure and fugacity becomes even larger at higher overpotentials (or fugacities) which is further demonstrated by plotting the fugacity coefficient ϕ (see Figure 10a). For overpotentials (fugacities) < 130 mV (<10^3^ bar) ϕ ≈ 1 results, indicating ideal gas behavior. However, at higher overpotentials (fugacities), ϕ increases drastically which reflects that at these overpotentials, the O_2_ gas formation and storage in our LSC films occurs at conditions far beyond ideal gas limitations.

Figure 10b displays the oxygen concentration as a function of the fugacity and the corresponding electrode overpotential at 460 °C. The chemical capacitance  is determined by the derivative of the oxygen concentration  with respect to the fugacity  as shown in eq 16. This corresponds to the slope of the concentration curve in Figure 10b. Hence, the increasing slope of the concentration curve with a maximum at 140 mV directly reflects the increasing *C*_chem_ curve and the maximum peak values at similar overpotentials. Furthermore, the decreasing concentration slope at higher overpotentials explains the peak-shaped *C*_chem_ curve with decreasing values beyond the maximum.

## 5 Conclusion

The chemical capacitance of LSC thin film microelectrodes with different microstructures was analyzed upon varying anodic DC voltages. In the case of dense and porous electrodes (open porosity), the chemical capacitance decreased with increasing overpotential in accordance with literature due to the decrease of the oxygen vacancy concentration. However, porous electrodes with a dense capping layer (closed porosity) exhibited an increase of the chemical capacitance with extremely high peak values  8000 F/cm^3^ at anodic overpotentials above 100 mV. In addition, it was shown that a higher measurement temperature leads to a shift of the capacitive peak to higher overpotentials.

Since this novel capacitive effect requires closed porosity, we considered the formation of high pressure oxygen in closed pores and calculated the corresponding chemical capacitance according to the Soave–Redlich–Kwong real gas equation. The thus calculated capacitance curve agrees very well with the experimental data at moderate overpotentials. Moreover, the real gas behavior explains the unexpected decrease of the chemical capacitance with increasing overpotential beyond the maximum despite increasing oxygen concentration in the closed pores. Therefore, we conclude that the formation of high pressure oxygen in closed pores is responsible for the observed peak of the chemical capacitance upon anodic polarization. This capicitive peak was reproducibly found even when measuring one electrode several times, which indicates that closed pores largely withstood gas pressures of ∼10^4^ bar.

More specifically the present study shows the following:

- The chemical capacitance of LSC thin film electrodes can be used to observe and quantify oxygen gas pressures in closed pores upon anodic polarization in synthetic air. This capacitive effect can also be of importance for SOEC applications. Whenever some sort of closed porosity occurs in such SOEC anodes or at the anode/electrolyte interface, either due to degradation phenomena or simply because of the configuration of the cell (i.e., current collector or interconnect on top of the electrode), high pressure oxygen will form and lead to high mechanical load.
- Analyzing the chemical capacitance of an electrode in SOEC mode could be used for detecting any kind of closed porosity at an early stage and thus may prevent destructive processes due to high pressure build-up. Since even ratios of closed porosity to bulk volume in the order of 10^–4^ should be detectable, this method might be used as a nondestructive measurement tool during SOEC operation.
- The formation of high pressure oxygen in closed pores corresponds to extremely high oxygen densities approaching molar volumes in the 30 cm^3^/mol range. The closed pores thus act as kind of nanovessels which can withstand pressures in the gigapascal range. Accordingly, determining mechanical gas pressures on the anode side of SOECs requires the application of real gas equations and fugacities.
- Closed pores in thin films can act as a chemical oxygen storage with capacitances  8000 F/cm^3^ or  400 mAhg^-1^V^-1^ and thus may be technologically interesting.

## Supporting Information Available

Fitting results of *R*_s_, *Q*, and *n* according to the equivalent circuit used for the chemical capacitance analysis (PDF)
