# Supplementary material for: Formation and Detection of High-Pressure Oxygen in Closed Pores of La0.6Sr0.4CoO3−δ Solid Oxide Electrolysis Anodes
Source: ACS Appl Energy Mater. 2022 Jun 23;5(7):8324–35. doi: 10.1021/acsaem.2c00888 (PMC9326814; doi:10.1021/acsaem.2c00888)
Supplement: Supplementary file 1 — ae2c00888_si_001.pdf [file ae2c00888_si_001.pdf]

Supporting Information: Formation and Detection of High-pressure Oxygen in Closed Pores of $La_{0.6}Sr_{0.4}CoO_{3-\delta}$ Solid Oxide Electrolysis Anodes

Martin Krammer,[*,†] Alexander Schmid,[†] Matthäus Siebenhofer,[†,‡] Andreas Ewald Bumberger,[†] Christopher Herzig,[†] Andreas Limbeck,[†] Markus Kubicek,[†] and Juergen Fleig[†]

†*TU Wien, Institute of Chemical Technologies and Analytics, Getreidemarkt 9/164-EC, 1060 Vienna, Austria*

‡*Centre for Electrochemical Surface Technology GmbH, Viktor-Kaplan-Straße 2, 2700 Wiener Neustadt, Austria*

E-mail: martin.krammer@tuwien.ac.at

Tables S1-S3 show the fitting results of R_s, Q and n according to the equivalent circuit depicted in Figure 4 at different bias voltages U_{DC} for each sample type. For each fitting procedure $n \geq 0.92$, therefore the exponent values of 1/n are sufficiently close to 1 for the calculation of the chemical capacitance C_{chem}.

Table S1: Fitting results for the dense electrode at 460 °C.

U_{DC} (mV)	R_s (Ωcm^2)	Q (mF s^{n-1})	n ()
0	3.37×10^2	3.49×10^{-3}	0.983
20	3.85×10^2	3.07×10^{-3}	0.984
40	3.56×10^2	2.75×10^{-3}	0.984
60	2.94×10^2	2.54×10^{-3}	0.986
80	2.35×10^2	2.37×10^{-3}	0.987
100	1.98×10^2	2.23×10^{-3}	0.987
120	1.78×10^2	2.08×10^{-3}	0.990
140	1.75×10^2	1.93×10^{-3}	0.991
160	1.87×10^2	1.77×10^{-3}	0.993
180	2.17×10^2	1.61×10^{-3}	0.993
200	2.63×10^2	1.47×10^{-3}	0.991
220	3.34×10^2	1.35×10^{-3}	0.990
240	4.26×10^2	1.25×10^{-3}	0.982
260	5.44×10^2	1.18×10^{-3}	0.984
280	6.74×10^2	1.13×10^{-3}	0.981
300	7.95×10^2	1.12×10^{-3}	0.983

Table S2: Fitting results for the porous electrode at 460 °C.

U_{DC} (mV)	R_s (Ωcm^2)	Q (mF s^{n-1})	n ()
0	5.77	1.83×10^{-3}	0.932
20	4.60	1.70×10^{-3}	0.940
40	4.18	1.60×10^{-3}	0.947
60	4.36	1.59×10^{-3}	0.946
80	4.82	1.60×10^{-3}	0.941
100	5.36	1.60×10^{-3}	0.938
120	5.82	1.58×10^{-3}	0.951
140	6.45	1.56×10^{-3}	0.949
160	7.02	1.54×10^{-3}	0.951
180	7.76	1.52×10^{-3}	0.946
200	8.51	1.48×10^{-3}	0.941
220	9.28	1.43×10^{-3}	0.940
240	1.02	1.39×10^{-3}	0.938
260	1.11	1.37×10^{-3}	0.935
280	1.24	1.40×10^{-3}	0.920

Table S3: Fitting results for the porous/capped electrode at 460 °C.

U_{DC} (mV)	R_s (Ωcm^2)	Q (mF s^{n-1})	n ()
0	2.88×10^{1}	2.47×10^{-3}	0.953
20	3.50×10^{1}	2.41×10^{-3}	0.952
40	4.12×10^{1}	2.36×10^{-3}	0.951
60	4.53×10^{1}	2.32×10^{-3}	0.957
80	4.67×10^{1}	2.33×10^{-3}	0.961
100	4.64×10^{1}	2.53×10^{-3}	0.963
120	4.56×10^{1}	3.06×10^{-3}	0.968
140	4.44×10^{1}	4.42×10^{-3}	0.973
160	4.35×10^{1}	6.93×10^{-3}	0.976
180	4.31×10^{1}	1.05×10^{-2}	0.977
200	4.32×10^{1}	1.38×10^{-2}	0.984
220	4.45×10^{1}	1.49×10^{-2}	0.982
240	4.69×10^{1}	1.44×10^{-2}	0.985
260	5.06×10^{1}	1.25×10^{-2}	0.975
280	5.37×10^{1}	1.12×10^{-2}	0.992
300	5.93×10^{1}	9.61×10^{-3}	0.989
320	6.62×10^{1}	8.27×10^{-3}	0.988
340	7.24×10^{1}	7.22×10^{-3}	0.990
360	7.14×10^{1}	6.61×10^{-3}	0.986
380	6.31×10^{1}	6.68×10^{-3}	0.999
